# Tracking the mental health of home-carers during the first COVID-19 national lockdown: evidence from a nationally representative UK survey

**DOI:** 10.1017/S0033291721002555

**Published:** 2021-06-10

**Authors:** Elise Whitley, Kelly Reeve, Michaela Benzeval

**Affiliations:** 1 MRC/CSO Social and Public Health Sciences Unit, University of Glasgow, Berkeley Square, 99 Berkeley Street, Glasgow G3 7HR, UK; 2 Institute for Social and Economic Research, University of Essex, Wivenhoe Park, Colchester, Essex CO4 3SQ, UK

**Keywords:** Carers, COVID-19, inequalities, longitudinal, mental health

## Abstract

**Background:**

Unpaid carers who look after another member of their household (home-carers) have poorer mental health than the general population. The first COVID-19 national lockdown led to an increasing reliance on home-carers and we investigate the short- and longer-term impacts of lockdown on their mental health.

**Methods:**

Data from 9737 adult participants (aged 16+) from the UK Household Longitudinal Study (Understanding Society) were used to explore changes in 12-item General Health Questionnaire (GHQ-12) score between (a) pre-pandemic (2019) and early lockdowns (April 2020) and (b) early and later (July 2020) lockdowns.

**Results:**

GHQ-12 scores among home-carers were higher pre-lockdown and increased more than for non-carers from 2019 to April 2020 with further increases for home-carers compared with non-carers between April and July. Compared with respondents caring for a spouse/partner, those caring for a child under 18 had a particularly marked increase in GHQ-12 score between 2019 and April, as did those caring for someone with a learning disability. Home-carers of children under 18 improved from April to July while those caring for adult children saw a marked worsening of their mental health. Home-carers with greater care burden saw larger increases in GHQ-12 score from 2019 to April and from April to July, and increases through both periods were greater for home-carers who had formal help prior to lockdown but then lost it.

**Conclusions:**

The mental health of home-carers deteriorated more during lockdown than non-carers. Policies that reinstate support for them and their care-recipients will benefit the health of both vulnerable groups.

**What is already known on this topic**

• Carers have poorer mental health than the general population.
• Among carers who live with the care-recipient (home-carers), some subgroups have poorer mental health than others: female *v.* male; those who provide more hours of care and have been caring for longer; spousal carers compared with those caring for children (including adult children), parents, or other relationships; those caring for individuals whose impairment results in behavioural disturbances, than those who care for individuals with physical or long-term health conditions.

**What this study adds**

• In a large representative UK survey, the decline in mental health during lockdown was greater among home-carers than for the general population, and stayed poorer through to July, even as the general population's mental health recovered slightly.
• Compared with respondents who were caring for a spouse/partner, those caring for a child under 18 had a particularly marked increase in GHQ-12 score between 2019 and April 2020 while those caring for an adult child experienced a substantial decline in their mental health between the beginning and end of the first lockdown (April to July 2020).
• The increase in GHQ-12 in April from 2019 was highest among those caring for someone with a learning disability and lowest for those caring for someone with a problem related to old age.
• Home-carers who had a greater care burden, in terms of hours of care provided, or lost formal support during lockdown, had poorer mental health.

## Introduction

At the start of 2020 around 8.8 million adults in the UK were unpaid carers (Carers UK, 2019) supporting individuals, most commonly close family members, with disabilities, long-term health conditions, or needs related to old age. Around half were caring for someone living in the same household (home-carers) and half for someone living elsewhere. In spring 2020, the COVID-19 pandemic reached the UK and ‘lockdown’ measures were introduced by the UK government. On 23rd March 2020, the Prime Minister announced the ‘staying at home and away from others’ policy, with people only able to leave home for limited purposes (shopping for basic necessities, one form of daily exercise, medical need, or providing care or help for a vulnerable person) (UK Government, 2020; UK Government Cabinet Office, 2020). This policy stayed in force for 6 weeks, then relaxed to allow unlimited exercise, return to school for some pupils, non-essential shops re-opening, and people meeting outdoors. The major change in re-opening society came on 4th July, when the hospitality sector opened and two households could meet and stay overnight in the same place (The Health Foundation, 2020). Alongside lockdown restrictions, many non-COVID-19 medical and social care services were withdrawn, cancelled, or changed from face-to-face to remote contact. These restrictions led to an increasing reliance by those with disabilities or ill health on informal support, with home-carers taking a particularly important role. Results from a Carer's UK survey in April 2020 (Carers UK, 2020a) indicate that 70% of existing carers were providing more care during lockdown, with more than a third doing so as a result of changes to services. Moreover, a repeat survey in October 2020 (Carers UK, 2020b) suggests that an additional 4.5 million new carers were created by the pandemic.

Informal caring is widely recognised as a stressful experience (Carers UK, 2015; Hirst, 2005; Schulz & Sherwood, 2008; Schulz, Beach, Czaja, Martire, & Monin, 2020; Stansfield et al., 2014) characterised by sleeplessness, loneliness, isolation, and high levels of unpredictability, uncontrollability, and vigilance. It is therefore unsurprising that the mental (and physical) health of carers is poor compared to the general population (Cuijpers, 2005; Hirst, 2005; Pinquart & Sorensen, 2003; Schoenmakers, Buntinx, & Delepeleire, 2010; Schulz et al., 2020; Schulz & Sherwood, 2008; Schulz, O'Brien, Bookwala, & Fleissner, 1995; Shah, Wadoo, & Latoo, 2010; Smith et al., 2014; Stansfield et al., 2014). In addition, within carers, there are particular high-risk groups. Mental health is worse among female carers (Cuijpers, 2005; Hirst, 2005; Pinquart & Sorensen, 2003; Schoenmakers et al., 2010; Schulz et al., 2020; Schulz & Sherwood, 2008; Shah et al., 2010), carers who live with the care-recipient (Hirst, 2005; Schulz et al., 2020; Shah et al., 2010), carers who provide more hours of support and have been caring for longer (Hirst, 2005; Schoenmakers et al., 2010; Schulz et al., 2020; Schulz & Sherwood, 2008; Smith et al., 2014), and carers with limited social and professional support (Schulz et al., 1995, 2020). Results for age are mixed with some studies reporting poorer mental health in older carers and others suggesting worse outcomes among those who are younger (Pinquart & Sorensen, 2003; Schulz et al., 2020; Schulz & Sherwood, 2008). The relationship between carer and care-recipient may also impact carers' health, with spousal carers having worse mental health than those caring for children (including adult), parents, or other relationships (Hirst, 2005; Pinquart & Sorensen, 2003; Schoenmakers et al., 2010; Schulz et al., 2020; Schulz & Sherwood, 2008). In addition, those caring for individuals whose impairment results in behavioural disturbances, for example mental health problems, dementia or cognitive impairment, have worse mental health than those caring for individuals with physical or long-term health conditions (Cuijpers, 2005; Pinquart & Sorensen, 2003; Schoenmakers et al., 2010; Schulz et al., 1995, 2020; Schulz & Sherwood, 2008; Shah et al., 2010).

Quarantine measures such as those imposed during the pandemic can have a negative psychological impact (Brooks et al., 2020) and concerns have been raised about the specific impact of COVID-19 lockdown measures on the mental health of the general population (Gunnell et al., 2020) as well as particular vulnerable groups including care-recipients and their carers (Holmes et al., 2020). Emerging evidence demonstrates that population mental health worsened in the early stages of lockdown (Niedzwiedz et al., 2020) although this may have reversed in the longer term (Chandola, Kumari, Booker, & Benzeval, 2020). However, a number of carer-specific surveys have reported significant worsening of mental health both in the short (Carers UK, 2020*a*; Pavlopoulou, Wood, & Papadopoulos, 2020; Reaching Families, 2020) and longer term (Carers UK, 2020*b*), although they do not make direct comparisons with the experiences of non-carers. Additionally, results from the UK Household Longitudinal Study (UKHLS) (Gallagher & Wetherell, 2020) indicate that individuals providing wide-ranging unpaid care and support for someone outside their household saw a decline in their mental wellbeing at the start of lockdown, which was particularly marked among women and older carers.

A number of carer-specific surveys highlight concerns and difficulties faced by carers during lockdown (Carers UK, 2020a; Giebel et al., 2020; Pavlopoulou et al., 2020; Reaching Families, 2020). Many care-recipients have long-term health conditions or disabilities that place them in the ‘high-risk’ group for COVID-19 (Office for National Statistics, 2020*a*) and early reports of limited access to ventilators and intensive care treatment led to considerable distress among some carers (Pavlopoulou et al., 2020). Carers themselves are also more likely than the general population to have long-term health problems (Carers UK, 2019) and a key concern was what would happen to their care-recipient if they became ill (Carers UK, 2020*a*). The lack of personal protective equipment for paid care workers led to the withdrawal of services by providers or voluntary reduction by carers themselves (Carers UK, 2020*a*; Giebel et al., 2020). Accessing non-COVID-19 health care was problematic for many disabled and long-term ill individuals and their carers (Office for National Statistics, 2020*b*). In addition, day service closures and respite care withdrawal was particularly difficult for individuals with learning disabilities, autism or dementia and many carers reported a marked increase in challenging behaviours as a result of their care-recipient not understanding changes to circumstances or new rules to be followed (Carers UK, 2020*a*; Giebel et al., 2020). Many carers also reported significant difficulties accessing food and other supplies (Carers UK, 2020*a*; Reaching families, 2020). Priority access for particular groups, most commonly those who were shielding, was granted by many supermarkets and suppliers but this process was slow to start and did not extend to many of the most vulnerable groups, who were not at additional risk from COVID-19 but had mental health or cognitive difficulties and could not conform to social distancing measures but, equally, could not be left alone.

The current analysis builds on existing evidence to better understand the impact of COVID-19 and associated mitigation measures on the mental health of home-carers. Based on longitudinal data from UKHLS annual interview and COVID-19 monthly web surveys, we focus on contemporaneous caring status during lockdown and compare the mental health of home-carers *v.* non-carers (or those only providing care outside the home) pre-pandemic and early (April) and later (July) in the first lockdown. We acknowledge the wide range of experiences of home-carers and explore differences in mental health according to different carer characteristics (their relationship to the care-recipient, the nature of the recipient's health condition(s), and the caring burden and support received). We consider the following research questions: (i) how did the mental health of home-carers change during early and later lockdown relative to that of non-carers; (ii) how did the mental health of home-carers vary according to their different circumstances; and (iii) were differences among home-carers and between home-carers and non-carers explained by demographic differences?

## Methods

Data are from a substudy of the UKHLS, a longitudinal, nationally representative study of the UK population (Institute for Social and Economic Research, 2020). All adults from households that took part in wave 8 or 9 of the main survey were invited to take part in the COVID-19 April web survey (*N* = 42 330) (Institute for Social and Economic Research, 2021). The COVID-19 survey has been repeated monthly and in July 2020, a module was added on caring responsibilities within the household. This paper uses data from the April and July COVID-19 surveys, and pre-pandemic data from 2019 (taken from wave 10 or 11 of the main survey based on interview date) (University of Essex, 2020). Analyses are based on respondents who took part in all three.

Home-caring status in July was based on responses to the question: *Is there anyone living with you who is sick, disabled or elderly whom you look after or give special help to* (*e.g. a sick, disabled or elderly relative, husband, wife or friend etc.*). Those giving a positive response were identified as home-carers and asked how many people in the household they cared for and, if more than one, to focus on the main care-recipient. Additional information was collected on the care-recipient's condition (long-term health condition (excluding mental health); long-term mental health condition; learning disability or developmental disorder such as autism; physical disability; problem related to old age; other); relationship to the home-carer (dependent child(ren) under 18; adult child(ren); parents or grandparents, including in-laws; siblings; spouse or partner; friends; other relatives; someone else); the hours per week spent caring (respondents were asked how many hours a week they provided care but a large number selected broader options (‘varies under 20 h’, ‘varies 20 h or more’, ‘100 h or more/continuous’) and these were used as cut-offs for this variable); and any support they received from others in the household or from formal respite or support services such as day-care centres, school, college, or carers supporting them in the home. Comparable questions on caring for a person living elsewhere were not included in the survey.

Respondents' mental health at all three time points (2019, April 2020, and July 2020) was measured using the 12-item General Health Questionnaire (GHQ-12), a widely used measure of non-psychotic psychological distress designed to capture depressive and anxiety symptoms (Goldberg & Williams, 1988). Each item has four response categories on a Likert scale ranging from ‘not at all’ to ‘much more than usual’ (scored 0, 0, 1, 1). Scores are then summed to produce a score between 0 and 12, with higher scores indicating poorer mental health. Primary analyses are based changes in scores so that changes in symptoms could be captured across the whole scale.

Analyses use ordinary least squares regression models comparing changes in GHQ-12 score between (a) pre-pandemic (2019) and early lockdown (April) and (b) early and later (July) lockdown, with all models adjusted for GHQ-12 scores at ‘baseline’ [(a) 2019 and (b) April 2020]. Preliminary models considered all respondents and compared changes in home-carers *v.* non-carers. Additional models were restricted to home-carers and compared changes according to their different circumstances. All models were then adjusted for age group (<40, 41–70, 71+), sex, education (degree or higher *v.* A level or lower), and ethnicity (white British *v.* non-white British or other) to explore whether differences between home-carers and non-carers and between different groups of home-carers were driven by demographic differences.

Standard errors were adjusted to account for the clustered and stratified sample and models included inverse probability weights to take account of unequal selection probabilities into the study and differential nonresponse at each wave, including to the COVID-19 survey. These weights ensure the results are reliable estimates representative of the UK adult population living in private households (University of Essex, 2020). In supplementary analyses, models were repeated based on GHQ-12 caseness with respondents scoring 4+ defined as having probable common mental disorder (CMD).

## Results

April web-survey interviews were completed by 17 761 participants and July web-survey interviews by 13 754 participants, with 12 680 individuals taking part in both. Of these, 12 209 (96%) also took part in a survey in 2019. The current analyses include respondents with complete data available in 2019, April and July surveys. The weighted analytical sample is 9737. However, some participants had missing data on one or more covariate, and hence the weighted analytical sample for individual models varies between 9369 and 9614 depending on the covariates included.

Table 1 describes the basic characteristics of the analytical sample. The mean age of participants was 50.8 [standard deviation (s.d.) 15.8] and 5037 (52%) were female. The majority of respondents were white British (88%) and 42% held a degree or higher qualification. In total 565 (6%) of respondents self-identified as home-carers in July 2020. Compared to non-carers, home-carers were older (22% *v.* 16% aged 71+), more likely to be women (58% *v.* 52%), and less likely to hold a degree or higher qualification (36% *v.* 43%). The final column of Table 1 presents comparable data for the full UKHLS sample who took part in the most recent pre-COVID-19 wave for which all data are available [wave 9 (2017–2019), *N* = 25 696]. Respondents included in the analytic sample were somewhat older (reflecting the later data collection date), had higher educational qualifications, and were less likely to consider themselves to be home-carers than those in the full wave 9 survey.

**Table 1.** Characteristics of analytical sample (respondents who took part in all of 2019, April, and July COVID-19 surveys) *v.* all respondents who took part in wave 9

	Analytical sample			Wave 9
	Full sample (*N* = 9737)^a^	Home-carers (*N* = 565)	Non-carers (*N* = 9170)^a^	Full sample (*N* = 25 696)
Age [*n* (%)]				
16–40	3123 (32.1)	136 (24.0)	2987 (32.6)	9118 (35.5)
41–70	5063 (52.0)	305 (53.9)	4758 (51.9)	12 396 (48.2)
71+	1551 (15.9)	125 (22.1)	1425 (15.5)	4174 (16.2)
Missing				9 (0.0)
Gender [*n* (%)]				
Men	4661 (47.9)	240 (42.5)	4419 (48.2)	12 385 (48.2)
Women	5037 (52.1)	325 (57.5)	4747 (51.8)	13 311 (51.8)
Education [*n* (%)]				
Degree or higher	4117 (42.4)	203 (35.9)	3914 (42.9)	9711 (37.8)
A-level	2196 (22.6)	122 (21.6)	2074 (22.7)	5665 (22.0)
GCSE	2004 (20.7)	128 (22.6)	1876 (20.5)	5203 (20.2)
Other	812 (8.4)	68 (12.0)	744 (8.1)	2280 (8.9)
No qualification	571 (5.9)	45 (8.0)	526 (5.8)	2746 (10.7)
Missing				91 (0.4)
Ethnicity [*n* (%)]				
White British	8561 (87.9)	495 (87.5)	8066 (88.0)	22 289 (86.7)
White Irish	91 (0.9)	7 (1.2)	84 (0.9)	338 (1.3)
White Other	246 (2.5)	7 (1.2)	239 (2.6)	793 (3.1)
Mixed	145 (1.5)	5 (0.9)	140 (1.5)	327 (1.3)
Asian/Asian British	415 (4.3)	47 (8.3)	368 (4.1)	1116 (4.3)
Black/Black British	147 (1.5)	2 (0.4)	145 (1.6)	463 (1.8)
Other	43 (0.4)	3 (0.5)	40 (0.4)	111 (0.4)
Missing				257 (1.0)
Home-carer [*n* (%)]				
No	9170 (94.2)	–	–	23 750 (92.4)
Yes	565 (5.8)	–	–	1903 (7.4)
Missing				43 (0.2)
GHQ-12 score (mean (s.d.))				
2019		2.45 (3.59)	1.89 (3.14)	
April 2020		3.17 (3.54)	2.77 (3.34)	
July 2020		2.94 (3.95)	1.96 (3.27)	

a Subtotals may not add up due to missing values.

Mean GHQ-12 scores were higher among home-carers *v.* non-carers in 2019 (2.45 *v.* 1.89). By April 2020 GHQ-12 scores in both groups had risen before falling again in July 2020. However, although July GHQ-12 scores among non-carers were similar to those in 2019, scores among home-carers continued to be higher than those measured prior to lockdown.

Table 2 presents key characteristics of the 565 respondents who identified as home-carers in July 2020. Care-recipients' relationship with their carer were: spouse/partner (41%), child under 18 (15%), adult child (10%), parent/grandparent (17%), and other (6%). Just over 10% of carers reported caring for more than one person. Care-recipients were reported as having a long-term health condition (45%), mental health condition (14%), physical disability (31%), learning disability (23%), problems related to old age (23%), or other (8%). The majority of home-carers (73%) reported also being a home-carer in 2019. Around 43% of home-carers provided care for under 20 h a week with a further 40% providing 20–100 h and 18% providing over 100 h or continuous care. Most home-carers reported never receiving any state care/services before the pandemic (84%); however, 11% stated that they received these services before the pandemic but lost it and 6% reported that they still received it. The majority did not share care responsibilities in the household (65%).

**Table 2.** Characteristics of 565 home-carers who took part in all of 2019, April, and July COVID-19 surveys

	*n*^a^ (%)
Care recipient relationship to carer	
Spouse or partner	232 (41.4)
Child under 18	82 (14.6)
Adult child	58 (10.3)
Parent or grandparent	94 (16.8)
Other	33 (5.9)
Care for more than one person	62 (11.1)
Care recipient condition^b^	
Long term health condition	257 (45.4)
Mental health condition	79 (14.0)
Physical disability	176 (31.2)
Learning disability	127 (22.5)
Problems related to old age	131 (23.2)
Other	50 (8.0)
Carer in 2019	
Yes	413 (73.1)
No	152 (26.9)
Hours of care per week	
Under 20 h	230 (42.7)
20–100 h	213 (39.5)
Over 100 h	96 (17.8)
State care/services	
Never had	465 (83.7)
Had before lockdown and lost	58 (10.5)
Had before lockdown and still do	32 (5.8)
Share care responsibilities	
Yes	196 (34.8)
No	368 (65.2)

a Subtotals may not add up due to missing values.

b Total is more than 100% as question is ‘please select all that apply’.

Table 3 presents changes in GHQ-12 scores from (a) 2019 to April and (b) April to July in home-carers *v.* non-carers. Mean scores among home-carers increased slightly more than those for non-carers from 2019 to April [change in GHQ-12 score (95% confidence interval) in home-carers *v.* non-carers: 0.26 (−0.09 to 0.61)]. However, increases from April to July were more marked among home-carers when compared with non-carers [0.77 (0.32 to 1.23)]. Adjustment for age, sex, education, and ethnicity had little impact on these results.

**Table 3.** Difference (95% confidence interval) in GHQ-12 score from (a) 2019 to April 2020 and (b) April 2020 to July 2020 according to home-carer status

	Adjusted for baseline GHQ-12		Additionally adjusted for age, sex, ethnicity, and education	
Caring status	2019 to April	April to July	2019 to April	April to July
Non-carer	0.00	0.00	0.00	0.00
Home carer	0.26 (−0.09 to 0.61)	0.77 (0.32 to 1.23)	0.33 (−0.03 to 0.69)	0.79 (0.33 to 1.25)

Table 4 restricts attention to the subgroup of respondents who self-identified as home-carers (*N* = 565) and examines changes in GHQ-12 score according to their different caring characteristics. There was little evidence of a difference in GHQ-12 scores by home-carers' age. However, home-carers who were women, who held a degree or higher qualification, or who were not white British saw greater increases in GHQ-12 score between 2019 and April 2020 [1.13 (0.45 to 1.81) for women *v.* men; 0.25 (0.08 to 0.43) for degree *v.* lower; 0.33 (0.04 to 0.61) for white British *v.* non-white British/other]; these differences were much reduced in analyses of change in GHQ-12 score between April and July. Looking at the relationship between carer and care-recipient, compared with respondents who were caring for a spouse/partner, those caring for a child under 18 had a particularly marked increase in GHQ-12 score between 2019 and April [0.91 (0.30 to 1.52)] with those caring for ‘other’ household members having a less marked increase. These differences were much reduced in comparison of GHQ-12 scores from April to July (−0.28 (−0.44 to 0.12) and [−0.16 (−0.32 to 0.00)]; however, home-carers responsible for adult children had considerably greater increases in GHQ-12 score over this period [2.75 (2.42 to 3.08)]. Home-carers who cared for more than one person in the household had markedly higher increases in GHQ-12 scores in April [1.54 (1.23 to 1.86)] and the same was true comparing July with April scores [1.21 (0.77 to 1.65)]. In terms of the care-recipient's condition, the increase in GHQ-12 in April from 2019 was highest among those caring for someone with a learning disability [1.06 (0.66 to 1.46)] and lowest for those caring for someone with a problem related to old age [−0.56 (−0.81 to −0.31)]. Looking at change in GHQ-12 from April to July, those caring for someone with a learning disability continued to have the greatest increase [1.32 (1.03 to 1.60)]. However, increases were also marked for those caring for individuals with long term [0.57 (0.37 to 0.78)] and mental health conditions [0.57 (0.29 to 0.85)] and physical disabilities [0.70 (0.51 to 0.88)], while those caring for someone with a problem related to old age saw a relative decline in score [−0.66 (−0.93 to −0.38)]. There was very little difference in the changes in GHQ-12 scores in either period comparing existing and new home-carers. Home-carers who provided higher numbers of hours of care per week saw an increase in their GHQ-12 scores from 2019 to April and, more markedly, from April to July [e.g. relative to those providing <20 h, change from April to July: 0.49 (0.27 to 0.71) and 1.09 (0.77 to 1.41) for those providing 20 to 100 and 100+ hours, respectively]. Increases in GHQ-12 through both periods were greater for home-carers who had had help from state care and services prior to lockdown but then lost it [e.g. April to July change relative to those who had never had support: 2.04 (1.51 to 2.56)]. Home-carers who shared responsibilities with other household members had a somewhat greater increase in GHQ-12 from 2019 to April [0.34 (0.10 to 0.58)] but this was no longer evident when comparing April to July. Adjusting for age, sex, education, and ethnicity had very little effect on the differences between different groups of carers.

**Table 4.** Difference (95% confidence interval) in GHQ-12 score from (a) 2019 to April 2020 and (b) April 2020 to July 2020 according to home-carer characteristics (analysis based on 565 home-carers)

	Adjusted for baseline GHQ-12		Additionally adjusted for age, sex, ethnicity, and education	
	2019 to April	April to July	2019 to April	April to July
Age group				
<40	0.00	0.00	–	–
41 to 70	0.40 (−0.45 to 1.24)	0.18 (−0.85 to 1.20)		
71+	−0.53 (−1.39 to 0.33)	−0.36 (−1.06 to 0.34)		
Sex				
Male	0.00	0.00	–	–
Female	1.13 (0.45 to 1.81)	0.38 (−0.55 to 1.30)		
Education				
A-level or lower	0.00	0.00	–	–
Degree or higher	0.25 (0.08 to 0.43)	0.02 (−0.13 to 0.16)		
Ethnicity				
White British	0.00	0.00	–	–
Non-White/Other	0.33 (0.04 to 0.61)	0.19 (−0.06 to 0.43)		
Relationship of care recipient to carer				
Spouse/partner	0.00	0.00	0.00	0.00
Child under 18	0.91 (0.30 to 1.52)	−0.28 (−0.44 to 0.12)	0.80 (0.10 to 1.50)	−0.42 (−0.65 to −0.19)
Adult child	0.39 (0.12 to 0.66)	2.75 (2.42 to 3.08)	−0.09 (−0.35 to 0.17)	2.64 (2.30 to 3.00)
(Grand)Parent	0.01 (−0.35 to 0.37)	0.58 (0.26 to 0.90)	−0.30 (−0.70 to 0.11)	0.45 (0.11 to 0.79)
Other	−0.57 (−0.76 to −0.37)	−0.16 (−0.32 to 0.00)	−0.58 (−0.81 to −0.35)	−0.27 (−0.46 to −0.07)
More than one	1.54 (1.23 to 1.86)	1.21 (0.77 to 1.65)	1.35 (0.92 to 1.79)	1.02 (0.54 to 1.52)
Care recipient has long-term health condition				
No	0.00	0.00	0.00	0.00
Yes	−0.14 (−0.44 to −0.17)	0.57 (0.37 to 0.78)	0.01 (−0.32 to 0.33)	0.64 (0.44 to 0.84)
Care recipient has mental health condition				
No	0.00	0.00	0.00	0.00
Yes	0.35 (−0.02 to 0.72)	0.57 (0.29 to 0.85)	0.19 (−0.19 to 0.56)	0.55 (0.24 to 0.86)
Care recipient has learning disability				
No	0.00	0.00	0.00	0.00
Yes	1.06 (0.66 to 1.46)	1.32 (1.03 to 1.60)	0.95 (0.57 to 1.33)	1.29 (1.01 to 1.57)
Care recipient has physical disability				
No	0.00	0.00	0.00	0.00
Yes	−0.10 (−0.44 to 0.24)	0.70 (0.51 to 0.88)	−0.01 (−0.41 to 0.40)	0.71 (0.52 to 0.90)
Care recipient has problem related to old age				
No	0.00	0.00	0.00	0.00
Yes	−0.56 (−0.81 to −0.31)	−0.66 (−0.93 to −0.38)	−0.43 (−0.69 to −0.17)	−0.64 (−0.95 to −0.33)
Care recipient has other condition				
No	0.00	0.00	0.00	0.00
Yes	0.03 (−0.79 to 0.85)	−0.11 (−0.76 to 0.55)	−0.11 (−0.79 to 0.56)	−0.19 (−0.83 to 0.45)
Carer in 2019				
No	0.00	0.00	0.00	0.00
Yes	−0.01 (−0.62 to 0.60)	−0.24 (−0.99 to 0.50)	0.19 (−0.38 to 0.76)	−0.25 (−0.95 to 0.45)
Hours of caring per week				
<20	0.00	0.00	0.00	0.00
20 to 100	0.57 (0.31 to 0.83)	0.49 (0.27 to 0.71)	0.37 (0.10 to 0.64)	0.43 (0.19 to 0.68)
101+	0.67 (0.13 to 1.20)	1.09 (0.77 to 1.41)	0.53 (0.03 to 1.03)	1.10 (0.72 to 1.37)
State care/services				
Never had	0.00	0.00	0.00	0.00
Had and lost	0.70 (0.27 to 1.14)	2.04 (1.51 to 2.56)	0.50 (0.09 to 0.91)	1.99 (1.50 to 2.48)
Still have	0.09 (−1.44 to 1.45)	−0.22 (−0.56 to 0.13)	0.19 (−1.20 to 1.59)	−0.22 (−0.56 to 0.13)
Share caring responsibilities				
No	0.00	0.00	0.00	0.00
Yes	0.34 (0.10 to 0.58)	0.07 (−0.16 to 0.30)	0.50 (0.27 to 0.73)	0.06 (−0.19 to 0.31)

Results from sensitivity analyses using 4+ symptoms as indicative of CMD were similar to those reported above (online Supplementary Appendix Tables X1 and X2). However, unadjusted odds ratios suggest that, compared with spousal carers, carers of adult children in April and of parents or grandparents in July were 2.00 (1.64 to 2.47) and 1.59 (1.01 to 2.49) times more likely respectively to show signs of psychological distress. Additionally, in July, those caring for someone with a mental health or long-term condition were 1.70 (1.25 to 2.33) and 1.56 (1.27 to 1.91), times more likely respectively to show signs of psychological distress than those caring for someone without these conditions.

## Discussion

An extensive literature highlights the stressful nature of caring (Carers UK, 2015; Hirst, 2005; Schulz et al., 2020; Schulz & Sherwood, 2008; Stansfield et al., 2014), and there is good reason to suppose that carers were disproportionately affected by COVID-19 and associated lockdown measures, particularly given reduction in social contact and social/health services and increases in home-caring responsibilities. Carers and care-recipients have both been identified as vulnerable groups in terms of the mental health consequences of COVID-19 and associated mitigation measures (Holmes et al., 2020), with an urgent call for research to better understand their experiences. Consistent with existing evidence (Cuijpers, 2005; Hirst, 2005; Pinquart & Sorensen, 2003; Schoenmakers et al., 2010; Schulz et al., 1995, 2020; Schulz & Sherwood, 2008; Shah et al., 2010; Smith et al., 2014; Stansfield et al., 2014), we found that home-carers had worse mental health than non-carers prior to the pandemic. By April 2020 mental health had deteriorated in both groups but to a greater extent among home-carers and, while there was some improvement in mental health between April and July in both groups, this was more modest among home-carers. As expected (Carers UK, 2019) home-carers were older and more likely to be women than non-carers but differences in GHQ scores were not explained by these factors, education or ethnicity.

Pre-pandemic evidence identified a number of carer groups at particularly high risk for poor mental health outcomes, including those living with the care-recipient (Hirst, 2005; Schulz et al., 2020; Shah et al., 2010), female carers (Cuijpers, 2005; Hirst, 2005; Pinquart & Sorensen, 2003; Schoenmakers et al., 2010; Schulz et al., 2020; Schulz & Sherwood, 2008; Shah et al., 2010), those providing more hours of care and caring for longer (Hirst, 2005; Schoenmakers et al., 2010; Schulz et al., 2020; Schulz & Sherwood, 2008; Smith et al., 2014), and those receiving less professional support (Schulz et al., 1995, 2020). Among home-carers in the current analyses, some were particularly badly affected by lockdown and, in general, these findings were consistent with reported differences between carers pre-pandemic. For example, early declines in mental health in April were more marked in female carers, although these differences were no longer evident by July. Individuals caring for more than one person and for more hours had particularly marked declines in mental health in April compared with pre-pandemic levels with further declines by July and those who lost respite care also fared worse in April with a further marked worsening of mental health by July. Around a quarter of those self-identifying as home-carers in July had not done so prior to the pandemic but there was no evidence that this group fared better or worse than pre-existing carers.

During the first lockdown, parents of children under 18 had markedly greater declines in mental health in April when compared with spousal or other home-carers but by July this same group had seen an improvement compared with others. Findings pre-pandemic generally report spousal carers as having the poorest mental health (Hirst, 2005; Pinquart & Sorensen, 2003; Schoenmakers et al., 2010; Schulz et al., 2020; Schulz & Sherwood, 2008), although mothers of sick or disabled children have also been identified as being at higher risk of psychological distress (Hirst, 2005). The current finding of poorer mental health in parents specifically of children under 18 may reflect the impact of school closures and subsequent partial reopening. In contrast, the mental health of parents caring for adult children was not markedly worse than other home-carers in April but saw a substantial decline by July, suggesting that their negative experiences were continuing and, indeed, worsening as lockdown progressed. This is consistent with the continuing closure of day and respite services, in contrast to schools re-opening, and reports of increases in challenging behaviours in those with autism and learning difficulties, as well as ongoing difficulties in accessing food and other supplies (Carers UK, 2020*a*; Reaching Families, 2020).

Existing evidence also suggests that those caring for individuals with behavioural disturbances, for example mental health problems, dementia or cognitive impairment, have worse mental health than those caring for individuals with physical or long-term health conditions (Cuijpers, 2005; Pinquart & Sorensen, 2003; Schoenmakers et al., 2010; Schulz et al., 1995, 2020; Schulz & Sherwood, 2008; Shah et al., 2010). In the current analyses, respondents caring for individuals with learning disabilities had the greatest declines in mental health early in lockdown. The further exacerbation of their mental health may again reflect the sudden closure of schools and day services. Similarly, the withdrawal of respite care has been highlighted as a particular issue by those caring for individuals with learning difficulties or autism (Pavlopoulou et al., 2020; Reaching Families, 2020), whereas health services for those with physical health problems were more likely to continue, albeit in a reduced or limited form. In addition, carers for those with learning difficulties reported additional challenges during lockdown as a result of their care-recipient not understanding new, often restricted, circumstances or guidance on hygiene and social distancing (Carers UK, 2020*a*) and this may have added additional burden to already stressed individuals.

By July home-carers for all conditions other than those associated with old age had seen a marked decline in their mental health. This highlights the ongoing cumulative strain of caring under lockdown conditions arising from anxiety regarding infection among those caring for someone who was shielding (Office for National Statistics, 2020*a*) or who were shielding themselves (Carers UK, 2019), lack of personal protective equipment and service withdrawal (Carers UK, 2020*a*; Giebel et al., 2020), problems accessing non-COVID-19 health care (Office for National Statistics, 2020*b*), and difficulties accessing food and other essential supplies (Carers UK, 2020*a*; Reaching Families, 2020). It has been suggested that individuals with pre-existing mental health problems might have been particularly vulnerable to COVID-19 mitigation measures (Brooks et al., 2020; Gunnell et al., 2020; Holmes et al., 2020) as well as disruptions to mental health services, particularly those based in the community (World Health Organisation, 2020). Consistent with this, our results suggest that home-carers for those with mental health problems, a group already known to be under significant stress, had particularly poor mental health themselves by July.

The current analyses are based on a large longitudinal dataset including detailed questions regarding respondents' caring experiences during lockdown and use of a validated mental health measure in repeated waves before and throughout the lockdown period. However, there are also some limitations. Given COVID-19 surveys were conducted on the web, they exclude those without internet access, who are generally older, have more health problems, and less education (Benzeval et al., 2021). Inverse probability weights have been demonstrated to reduce these biases, but some minor differences remain. In particular, comparison with caring status in pre-pandemic waves suggests that home-carers may still have been under-represented in the COVID-19 surveys. It is plausible that home-carers with the greatest burden were less likely to participate in the COVID-19 surveys and our results comparing the mental health of carers with non-carers may therefore be under-estimates of the real effect. In contrast to previous study (Gallagher & Wetherell, 2020), we considered caring status during rather than before lockdown as the pandemic created new caring roles (Carers UK, 2020*b*). However, this was measured in July, and so may not accurately reflect circumstances in April although the error is likely to be small and results for new and pre-existing home-carers were almost identical. Although detailed data were available on the caring experiences of respondents during the pandemic these were not exhaustive, for example we lacked information regarding the type of care provided. In addition, responses required the use of quite broad categories for some variables, for example hours of care provided, although these coincide with previous definitions of ‘high intensity’ caregiving (Hirst, 2005; Schulz et al., 2020; Smith et al., 2014). In addition, we only consider respondents caring for another household member as comparable information was not available for those providing care for someone living elsewhere. Our results are therefore not generalisable to non-resident carers who are likely to have faced different challenges to home-carers. Mental health was assessed using GHQ-12, which is a widely used, validated measure (Goldberg & Williams, 1988), but lacks the specificity of more targeted depression scales. However, this generalised measure allowed us to capture a wider range of mental health and, importantly, its repeated inclusion in all UKHLS waves enabled us to track changes over time. Finally, we consider mental health at the beginning of the first lockdown and as it progressed. However, given further national lockdowns, it will be important to revisit these issues to investigate the short- and longer-term impacts of repeated restrictions and reduced support on the mental health of carers.

## Conclusion

Informal home-carers have been described as ‘the forgotten health-care workers during the COVID-19 pandemic’ (Chan et al., 2020). Our results clearly demonstrate that the mental health of home-carers, which was already poor pre-pandemic (Cuijpers, 2005; Hirst, 2005; Pinquart & Sorensen, 2003; Schoenmakers et al., 2010; Schulz et al., 1995, 2020; Schulz & Sherwood, 2008; Shah et al., 2010; Smith et al., 2014; Stansfield et al., 2014), has been disproportionately affected by COVID-19 and associated mitigation measures. Responses to the pandemic have largely focussed on infection control but there is also growing recognition of the need to support mental health. Our study shows this is particularly an issue for carers and, as the pandemic continues, better policies that support the mental health of the population in general and carers in particular are required.

The restarting of relevant health and social care services for those requiring care should be a priority to reduce the burden on informal home-carers, with consideration given to how to support carers of those with conditions such as dementia, learning disabilities, and mental illness, whose mental health has been particularly badly affected. Assessment should be made of increased need due to COVID-19, and temporary care provided by family members during lockdown should not be regarded as a long-term solution or replacement for formal care that was withdrawn. Data should be collected to better understand service withdrawal and the impact this had on care-recipients and their carers. In addition, carers should be recognised as a vulnerable group, regardless of the diagnosis of the person they care for and, in the event of further restrictions, be given priority access to food and other essential services.

There are millions of unpaid home-carers in the UK who provide vital support to members of their household. Several decades of research has highlighted the stressful nature of caring and provides robust evidence of worse mental health among carers compared with non-carers (Cuijpers, 2005; Hirst, 2005; Pinquart & Sorensen, 2003; Schoenmakers et al., 2010; Schulz et al., 1995, 2020; Schulz & Sherwood, 2008; Shah et al., 2010; Smith et al., 2014; Stansfield et al., 2014). This evidence has led to recommendations that caregiving be recognised as a specific public health issue (Carers UK, 2019; Hirst, 2005) and that the NHS should develop policies and protocols to identify unpaid carers through primary care and other relevant settings and the health needs of carers be subject to regular review alongside those of the person they care for. Indeed, emerging evidence suggests that approaches that consider the health of both carers and care-recipients simultaneously may be particularly effective (Schulz et al., 2020). However, the experiences of carers suggest that these recommendations have not been widely adopted, with a survey carried out by Carers UK in 2019 indicating that over three quarters of carers were concerned about the impact of caring on their own health (Carers UK, 2019). Our findings, along with those from other COVID-19-specific research, shine a spotlight on the challenges faced by this neglected group. The post-COVID-19 restart of health and social services provides an ideal opportunity to recognise the vital role of individuals who provide care for sick and disabled household members, to identify the specific health care needs of unpaid carers, and to consider how best to support them and ensure that their mental and physical health is a priority.

## Supplementary material

For supplementary material accompanying this paper visit http://dx.doi.org/10.1017/S0033291721002555. click here to view supplementary material
