# Supplementary material for: Tracking the mental health of home-carers during the first COVID-19 national lockdown: evidence from a nationally representative UK survey
Source: Psychol Med. 2021 Jun 10:1–10. doi: 10.1017/S0033291721002555 (PMC8245331; doi:10.1017/S0033291721002555)
Supplement: Supplementary file 1 [file S0033291721002555sup001.docx]

***Appendix tables***

Table X1: Odds ratio (95% confidence interval) for probable common mental disorder in (a) April 2020 and (b) July 2020 according to home-carer status

| Caring status | Adjusted for baseline GHQ-12 | | Additionally adjusted for age, sex, ethnicity and education | |
| --- | --- | --- | --- | --- |
|  | April | July | April | July |
|  |  |  |  |  |
| Non-carer  Home carer | 1.00  1.40 (1.12, 1.74) | 1.00  1.59 (1.19, 2.13) | 1.00  1.51 (1.20, 1.90) | 1.00  1.66 (1.24, 2.22) |
|  |  |  |  |  |

Table X2: Odds ratio (95% confidence interval) for probable common mental disorder in (a) April 2020 and (b) July 2020 according to home-carer characteristics (analysis based on 565 home-carers)

|  | | Adjusted for baseline GHQ-12 | | | | | | Additionally adjusted for age, sex, ethnicity and education | | |
| --- | --- | --- | --- | --- | --- | --- | --- | --- | --- | --- |
|  | April | | | | July | | | | April | July |
|  |  | | | |  | | | |  |  |
| Age group |  | | | |  | | | |  |  |
| <40  41-70  71+ | 1.00  1.15 (0.66, 2.00)  0.62 (0.32, 1.20) | | | | 1.00  1.21 (0.65, 2.26)  0.73 (0.39, 1.38) | | | | - | - |
|  |  | | | |  | | | |  |  |
| Sex |  | | | |  | | | |  |  |
| Male  Female | 1.00  2.41 (1.50, 3.88) | | | | 1.00  1.53 (0.86, 2.72) | | | | - | - |
|  |  | | | |  | | | |  |  |
| Education |  | | | |  | | | |  |  |
| A-level or lower  Degree or higher | 1.00  0.47 (0.30, 0.75) | | | | 1.00  1.12 (0.65, 1.91) | | | | - | - |
|  |  | | | |  | | | |  |  |
| Ethnicity |  | | | |  | | | |  |  |
| White British  Non-White/Other | 1.00  1.16 (0.72, 1.87) | | | | 1.00  0.98 (0.59, 1.61) | | | | - | - |
|  |  | | | |  | | | |  |  |
| Relationship of care recipient to carer | | | |  |  | | | |  |  |
| Spouse/partner  Child under 18  Adult child  (Grand)Parent  Other  More than one | 1.00  1.65 (1.19, 2.28)  2.00 (1.64, 2.47)  0.95 (0.66, 1.36)  0.89 (0.74, 1.07)  2.69 (1.88, 3.85) | | | | 1.00  0.79 (0.62, 1.00)  3.37 (2.67, 4.25)  1.59 (1.01, 2.49)  1.04 (0.86, 1.26)  2.80 (1.91, 4.10) | | | | 1.00  1.40 (0.93, 2.12)  1.46 (1.19, 1.78)  0.66 (0.45, 0.97)  0.87 (0.70, 1.08)  2.24 (1.42, 3.53) | 1.00  0.71 (0.56, 0.91)  2.76 (2.14, 3.54)  1.52 (0.92, 2.50)  0.92 (0.74, 1.15)  2.60 (1.68, 4.01) |
|  |  | | | |  | | | |  |  |
| Care recipient has long term health condition | | | | | |  |  | |  |  |
| No  Yes | 1.00  0.79 (0.63, 1.00) | | | | 1.00  1.56 (1.27, 1.91) | | | | 1.00  0.87 (0.67, 1.13) | 1.00  1.66 (1.35, 2.03) |
|  |  | | | |  | | | |  |  |
| Care recipient has mental health condition | | | | | |  |  | |  |  |
| No  Yes | 1.00  2.11 (1.54, 2.89) | | | | 1.00  1.70 (1.25, 2.33) | | | | 1.00  1.86 (1.32, 2.63) | 1.00  1.66 (1.19, 2.30) |
|  |  | | | |  | | | |  |  |
| Care recipient has learning disability | | | |  |  | | | |  |  |
| No  Yes | 1.00  2.16 (1.71, 2.72) | | | | 1.00  2.09 (1.67, 2.61) | | | | 1.00  2.11 (1.64, 2.71) | 1.00  2.00 (1.57, 2.54) |
|  |  | | | |  | | | |  |  |
| Care recipient has physical disability | | | |  |  | | | |  |  |
| No  Yes | 1.00  0.90 (0.70, 1.17) | | | | 1.00  1.39 (1.11, 1.73) | | | | 1.00  0.87 (0.65, 1.14) | 1.00  1.36 (1.09, 1.70) |
|  |  | | | |  | | | |  |  |
| Care recipient has problem related to old age | | | | | |  |  | |  |  |
| No  Yes | 1.00  0.71 (0.57, 0.89) | | | | 1.00  0.83 (0.55, 1.26) | | | | 1.00  0.78 (0.61, 0.99) | 1.00  0.91 (0.57, 1.47) |
|  |  | | | |  | | | |  |  |
| Care recipient has other condition | | | |  |  | | | |  |  |
| No  Yes | 1.00  0.69 (0.47, 1.00) | | | | 1.00  0.80 (0.36, 1.78) | | | | 1.00  0.63 (0.47, 0.85) | 1.00  0.72 (0.35, 1.50) |
|  |  | | | |  | | | |  |  |
| Carer in 2019 |  | | | |  | | | |  |  |
| No  Yes | 1.00  1.06 (0.69, 1.64) | | | | 1.00  0.91 (0.52, 1.60) | | | | 1.00  1.31 (0.83, 2.06) | 1.00  0.90 (0.53, 1.53) |
|  |  | | | |  | | | |  |  |
| Hours of caring per week | | |  | |  | | | |  |  |
| <20  20-100  101+ | 1.00  1.59 (1.29, 1.96)  1.33 (0.94, 1.90) | | | | 1.00  1.57 (1.17, 2.10)  2.37 (1.75, 3.23) | | | | 1.00  1.44 (1.15, 1.82)  1.20 (0.84, 1.70) | 1.00  1.44 (1.09, 1.90)  2.20 (1.61, 3.01) |
|  |  | | | |  | | | |  |  |
| State care/services |  | | | |  | | | |  |  |
| Never had  Had and lost  Still have | 1.00  1.56 (1.21, 2.02)  0.80 (0.42, 1.50) | | | | 1.00  2.86 (1.56, 5.22)  0.81 (0.44, 1.50) | | | | 1.00  1.36 (1.02, 1.80)  0.96 (0.51, 1.81) | 1.00  2.70 (1.62, 4.51)  0.83 (0.48, 1.43) |
|  |  | | | |  | | | |  |  |
| Share caring responsibilities | | |  | |  | | | |  |  |
| No  Yes | 1.00  1.16 (0.96, 1.42) | | | | 1.00  1.01 (0.78, 1.30) | | | | 1.00  1.28 (1.05, 1.55) | 1.00  1.04 (0.81, 1.33) |
|  |  | | | |  | | | |  |  |
